# A Novel Ambroxol-Derived Tetrahydroquinazoline with a Potency against SARS-CoV-2 Proteins

**DOI:** 10.3390/ijms24054660

**Published:** 2023-02-28

**Authors:** Alena I. Krysantieva, Julia K. Voronina, Damir A. Safin

**Affiliations:** 1 Institute of Chemistry, University of Tyumen, Volodarskogo Str. 6, 625003 Tyumen, Russia; 2 N.S. Kurnakov Institute of General and Inorganic Chemistry of the Russian Academy of Sciences, Leninsky prospekt 31, GSP-1, 119991 Moscow, Russia; 3 Scientific and Educational and Innovation Center for Chemical and Pharmaceutical Technologies, Ural Federal University Named after the First President of Russia B.N. Yeltsin, 620002 Ekaterinburg, Russia

**Keywords:** ambroxol, tetrahydroquinazoline, synthesis, crystal structure, NMR, X-ray, molecular docking, molecular dynamics, SARS-CoV-2, COVID-19

## Abstract

We report synthesis of a novel 1,2,3,4-tetrahydroquinazoline derivative, named 2-(6,8-dibromo-3-(4-hydroxycyclohexyl)-1,2,3,4-tetrahydroquinazolin-2-yl)phenol (**1**), which was obtained from the hydrochloride of 4-((2-amino-3,5-dibromobenzyl)amino)cyclohexan-1-ol (ambroxol hydrochloride) and salicylaldehyde in EtOH. The resulting compound was produced in the form of colorless crystals of the composition **1**∙0.5EtOH. The formation of the single product was confirmed by the IR and ^1^H spectroscopy, single-crystal and powder X-ray diffraction, and elemental analysis. The molecule of **1** contains a chiral tertiary carbon of the 1,2,3,4-tetrahydropyrimidine fragment and the crystal structure of **1**∙0.5EtOH is a racemate. Optical properties of **1**∙0.5EtOH were revealed by UV-vis spectroscopy in MeOH and it was established that the compound absorbs exclusively in the UV region up to about 350 nm. **1**∙0.5EtOH in MeOH exhibits dual emission and the emission spectra contains bands at about 340 and 446 nm upon excitation at 300 and 360 nm, respectively. The DFT calculations were performed to verify the structure as well as electronic and optical properties of **1**. ADMET properties of the *R*-isomer of **1** were evaluated using the SwissADME, BOILED-Egg, and ProTox-II tools. As evidenced from the blue dot position in the BOILED-Egg plot, both human blood–brain barrier penetration and gastrointestinal absorption properties are positive with the positive PGP effect on the molecule. Molecular docking was applied to examine the influence of the structures of both *R*-isomer and *S*-isomer of **1** on a series of the SARS-CoV-2 proteins. According to the docking analysis results, both isomers of **1** were found to be active against all the applied SARS-CoV-2 proteins with the best binding affinities with Papain-like protease (PLpro) and nonstructural protein 3 (Nsp3_range 207–379-AMP). Ligand efficiency scores for both isomers of **1** inside the binding sites of the applied proteins were also revealed and compared with the initial ligands. Molecular dynamics simulations were also applied to evaluate the stability of complexes of both isomers with Papain-like protease (PLpro) and nonstructural protein 3 (Nsp3_range 207–379-AMP). The complex of the *S*-isomer with Papain-like protease (PLpro) was found to be highly unstable, while the other complexes are stable.

## 1. Introduction

The history of mankind is known as a constant fight against problems of health. Of these problems, diseases turned to become pandemics are the most crucial and fatal since they become global and their consequences often cause hard-to-recover human and economic losses. The situation becomes even more crucial upon emergence of new diseases, for which neither efficient drugs nor therapy are known; thus, mankind is constantly on the bloody warpath against diseases. This “war”, obviously, requires the continuous design and synthesis of new molecules with desirable biological and therapeutic properties, as well as the efficient production of novel drugs based on them.

During the last three years, mankind has been faced with one of the most dangerous viruses, namely severe acute respiratory syndrome-related coronavirus 2 (SARS-CoV-2). This virus is a strain of coronavirus that causes coronavirus disease 2019 (COVID-19), which was announced as a pandemic by the World Health Organization (WHO) in March 2020. To date, as of February 2023, about 755 million infections were confirmed with more than 6.8 million deaths [1]. The situation with COVID-19 still remains complicated due new strains, of which variants of concern are alpha, beta, gamma, delta, and omicron; thus, drugs against COVID-19 are of particular value.

There are no doubts that heterocyclic compounds are of great importance and play a pivotal role in nature. Suffice it to mention cytosine, guanine, adenine, and thymine, which are nitrogen-containing heterocyclic compounds, all four being nucleobases for deoxyribonucleic acid (DNA). The latter is fundamental for many viruses and all organisms; furthermore, an overwhelming majority of drugs are constructed from heterocyclic compounds. As such, nowadays, a great number of heterocyclic compounds with a pronounced pharmacological activity have been developed and are available on the market [2,3,4,5,6,7,8]. It was also reported that the heterocyclic fragments can serve as valuable and important resources for the development of coronaviruses’ treatment strategies and therapy [9,10]; furthermore, natural products containing heterocycles are also of interest as antiviral agents [11].

Of a great variety of heterocyclic compounds, quinazolines, containing fused benzene and pyrimidine six-membered rings, are a large family with biological properties of particular importance [12,13]. Of a variety of quinazoline derivatives [14,15,16], some important drugs can be highlighted such as: *afatinib* and *gefitinib* for treatment of non-small-cell carcinoma [17,18], *lapatinib* for treatment of advanced-stage or metastatic breast cancer [19], and *erlotinib*, an antitumor agent [20].

Partial hydrogenation of quinazoline leads to 1,2,3,4-tetrahydroquinazoline, where the pyrimidine fragment turns to 1,2,3,4-tetrahydropyrimidine, which synthesis was first reported by Pietro Biginelly in 1893 [21]. Since that time, 1,2,3,4-tetrahydropyrimidine and its derivatives have attracted attention of scientists from different fields due to both their efficient synthetic approaches [22,23,24,25,26], and biological and chemotherapeutic activities [27,28,29,30]. Furthermore, the novel 1,2,3,4-tetrahydropyrimidine derivative as inhibitor of SARS-CoV-2 was reported recently [31]. Thus, 1,2,3,4-tetrahydroquinazoline derivatives are of potential interest for the therapy of COVID-19.

**Figure 1:**
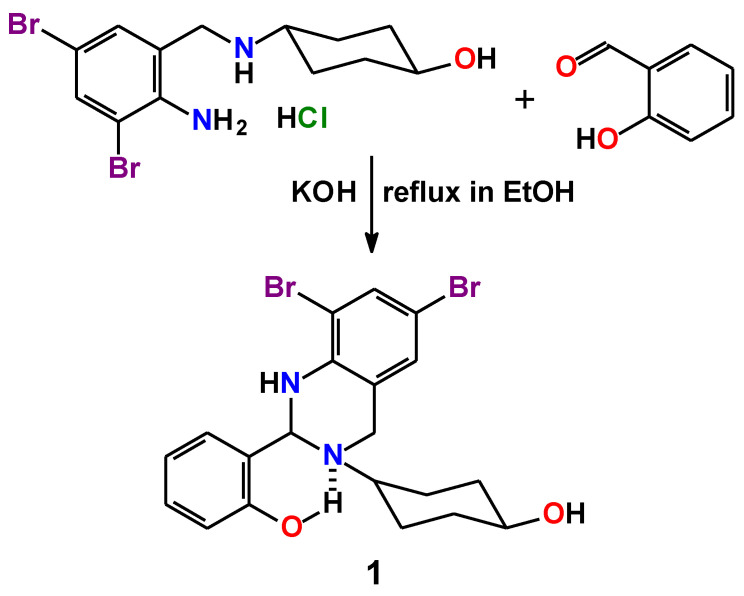
Synthesis of **1**.

With all this in mind, as well as in continuation of our ongoing interest in the chemistry of nitrogen-containing six-membered rings [32,33,34,35,36,37,38,39] and in in silico studies of bioactive compounds [40,41,42,43,44,45,46,47,48,49,50,51,52,53,54,55], we have directed our attention to a novel 1,2,3,4-tetrahydroquinazoline derivative, named 2-(6,8-dibromo-3-(4-hydroxycyclohexyl)-1,2,3,4-tetrahydroquinazolin-2-yl)phenol (**1**), which was obtained from the hydrochloride of 4-((2-amino-3,5-dibromobenzyl)amino)cyclohexan-1-ol, which is commonly known under its trademark ambroxol, and salicylaldehyde and is constructed from the tetrahydroquinazoline, phenylene, and cyclohexylene rings (Figure 1). Theoretical density-functional-theory (DFT)-based calculations were applied to **1** to reveal its electronic and optical properties. Bioavailability, druggability, as well as absorption, distribution, metabolism, excretion, and toxicity (ADMET) properties of **1** were evaluated using a set of online tools. Using an in silico molecular docking method, we have explored the binding modes and interactions of **1** with binding sites of a series of the SARS-CoV-2 proteins.

## 2. Results and Discussion

Recently, it was reported about using ambroxol, which is known for its mucolytic and expectorant properties, as potential pharmacotherapy for SARS-CoV-2 [56]. We have also applied detailed in silico studies to ambroxol and, according to the molecular docking results, it was revealed that the molecule of ambroxol interacts much more efficiently with a series of the studied SARS-CoV-2 proteins in comparison to Favipiravir [48]; furthermore, in 1990 Lai et al. reported on substituted salicylaldehyde Schiff bases as new antiviral agents against coronavirus [57]. All this encouraged us to produce a salicylaldehyde Schiff base from ambroxol with the aim to study possible tautomerism of the resulting product as well as its tautomer-dictated ADMET properties and antiviral activity against a series of the SARS-CoV-2 proteins using computational approaches. As such, we have involved ambroxol hydrochloride into a condensation reaction with salicylaldehyde in ethanol; however, due to the presence of a secondary amine group in the structure of ambroxol, the resulting imine function, being in a close proximity, was further reacted with this amine nitrogen atom, yielding a cyclization product of the novel 1,2,3,4-tetrahydroquinazoline derivative, named 2-(6,8-dibromo-3-(4-hydroxycyclohexyl)-1,2,3,4-tetrahydroquinazolin-2-yl)phenol (**1**) (Figure 1). The resulting product was formed as colorless crystals, suitable for single-crystal X-ray diffraction, of the **1**∙0.5EtOH composition.

**Figure 2:**
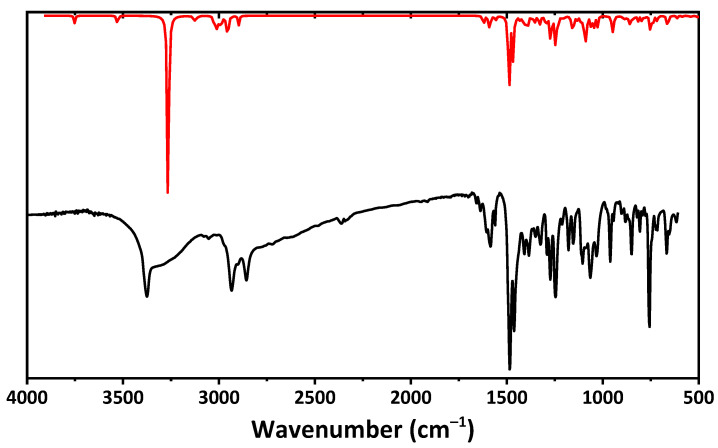
The experimental IR spectrum of **1**∙0.5EtOH recorded in a KBr pellet (black) and calculated IR spectrum of the *R*-isomer of **1** in gas phase (red), obtained using the DFT/B3LYP/6-311++G(d,p) method.

The IR spectrum of **1**∙0.5EtOH, recorded in the KBr pellet, contains broad and sharp bands at 3100–3600 cm^−1^ (Figure 2), corresponding to OH and NH groups. Low intense bands at about 3050 and 3080 cm^−1^ are due to CH stretching vibrations of the aromatic rings, while bands at 2790–3000 cm^−1^ correspond to CH stretching vibrations of the cyclohexane, methylene, and methine fragments. A set of bands at 1530–1675 cm^−1^ corresponds to C=C bending and N–H stretching. The most intense bands at 1460 and 1485 cm^−1^ were attributed to bending of the C–H and O–H functionalities. Notably, some contribution to the experimental IR spectrum is also expected from EtOH, which is trapped in the crystal structure of **1**∙0.5EtOH (see discussion below).

**Figure 3:**
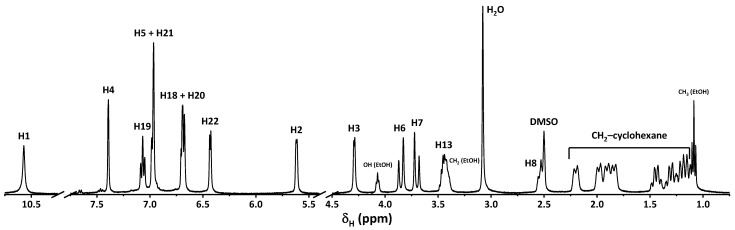
The ^1^H NMR spectrum of **1**∙0.5EtOH recorded in DMSO-*d*_6_.

The ^1^H NMR spectrum of **1**∙0.5EtOH recorded in DMSO-*d*_6_ contains a single set of peaks, corresponding to both **1** and EtOH (Figure 3). Particularly, peaks of the cyclohexane CH_2_ protons were observed as a series of multiplets at 1.13–2.27 ppm, while the cyclohexane CH hydrogen atoms were found as two multiplets at about 2.53 and 3.44 ppm, both partially overlapping with the signals from the DMSO and EtOH methylene hydrogens, respectively. The NH and CH protons of the 1,2,3,4-tetrahydropyrimidine fragment were found as two doublets at 4.29 and 5.62 ppm, respectively, while the CH_2_ protons of the same fragment were shown as two doublets 3.70 and 3.85 ppm, respectively. The cyclohexanol and phenolic OH protons were revealed as a doublet and singlet at 6.43 and 10.57 ppm, respectively. Finally, a set of peaks at 6.60–7.43 ppm was assigned to aromatic protons.

**Figure 4:**
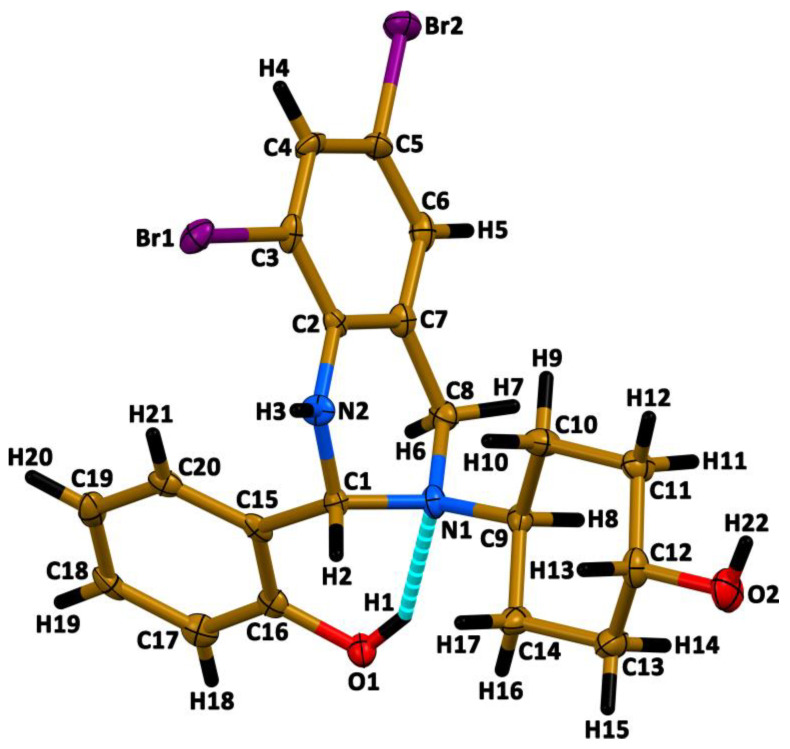
View of Molecule A in the crystal structure of **1**∙0.5EtOH. Molecule B and EtOH were omitted for clarity.

According to single-crystal X-ray diffraction, **1**∙0.5EtOH crystallizes in triclinic space group *P*-1, and the asymmetric unit cell contains two molecules, named Molecule A and Molecule B, and a half of the EtOH molecule. Notably, in Molecule B all the cyclohexane atoms, except for the carbon atom attached to the OH fragment, are disordered over two positions with a ratio of 49.5% to 50.5%. The solvent molecule is also disordered over two positions but with ratio of 48.8% to 51.2%. It should also be noted that the molecule of **1** contains a chiral carbon C1 (Figure 4) and the crystal structure of **1**∙0.5EtOH is a racemate. Bond lengths, and bond and dihedral angles in Molecule A and Molecule B are within the expected ranges (Table 1). Interestingly, the N2–C2 and O1–C16 bond lengths are about 0.06–0.07 Å shorter in comparison to the related N2–C1 and O2–C12 bonds (Table 1), which is explained by conjugation of the lone pairs of N2 and O1 with the π-system of the corresponding aromatic rings (Figure 4).

Both Molecule A and Molecule B in the crystal structure of **1**∙0.5EtOH are stabilized by an intramolecular hydrogen bond O1–H1∙∙∙N1, formed between the phenolic OH hydrogen atom and the nitrogen atom of the 1,2,3,4-tetrahydropyrimidine tertiary amine group (Figure 4, Table 2).

**Figure 5:**
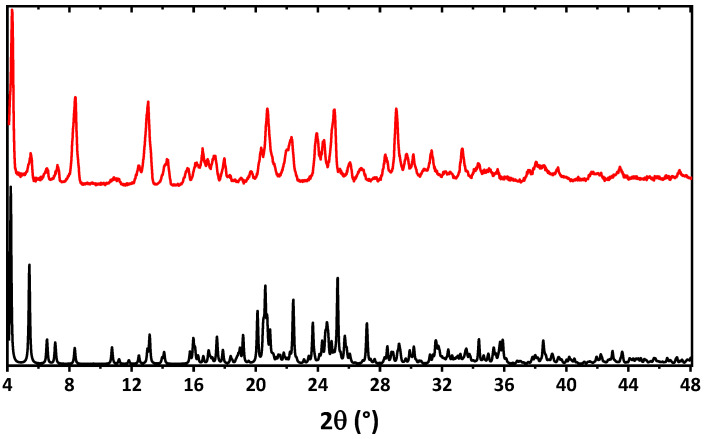
Calculated (black) and experimental (red) powder X-ray diffraction patterns of **1**∙0.5EtOH.

The bulk sample of **1**∙0.5EtOH was examined by means of powder X-ray diffraction analysis (Figure 5). The experimental X-ray powder pattern is in full agreement with the calculated powder pattern obtained from single-crystal X-ray diffraction, showing that the bulk material is free from phase impurities.

**Figure 6:**
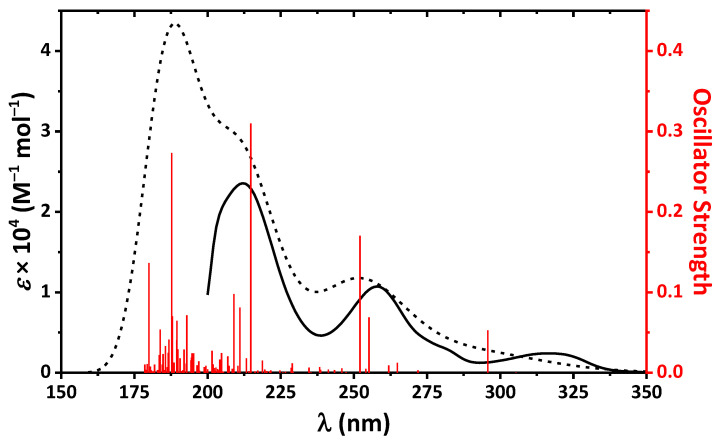
The experimental UV-vis spectrum of **1**∙0.5EtOH in MeOH (solid black) and calculated UV-vis spectrum of the *R*-isomer of **1** in gas phase (dashed black) together with oscillators (red), obtained using the DFT/B3LYP/6-311++G(d,p) method.

The absorption spectrum of **1**∙0.5EtOH in MeOH contains bands exclusively in the UV region up to about 350 nm with three clearly defined maxima at 212, 258, and 316 nm (Figure 6). The second band is accompanied with a low intense shoulder at about 280 nm (Figure 6).

**Figure 7:**
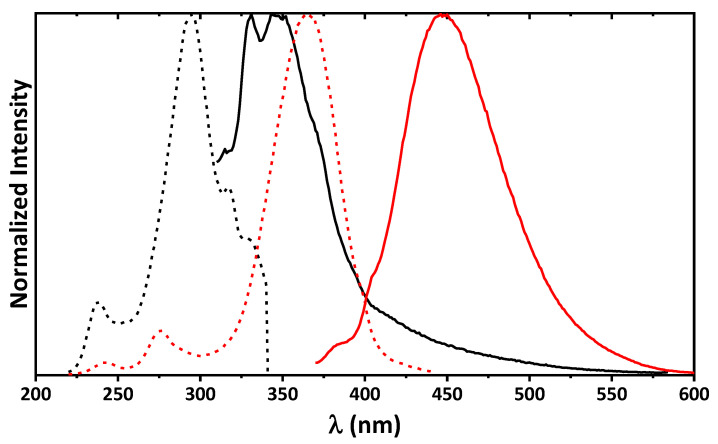
Normalized emission (solid black, λ_exc_ = 300 nm and solid red, λ_exc_ = 360 nm) and excitation (dashed black, λ_em_ = 340 nm and dashed red, λ_em_ = 450 nm) spectra of **1**∙0.5EtOH in MeOH.

**Figure 8:**
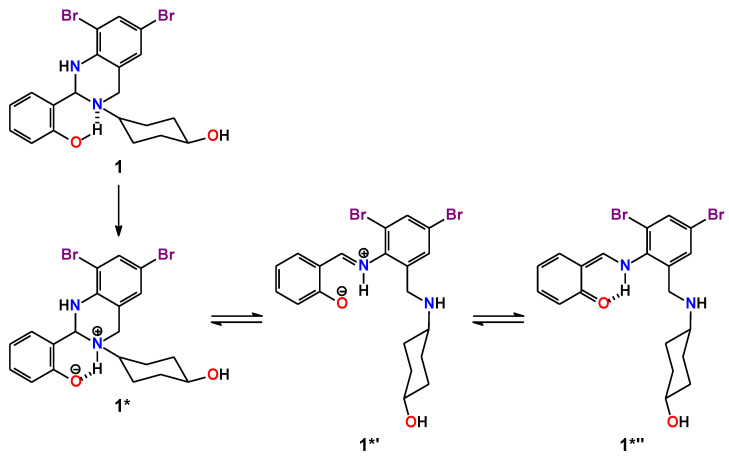
Plausible isomerization of **1** in the excited state.

Surprisingly, **1**∙0.5EtOH in MeOH exhibits dual emission and the emission spectra contains bands at about 340 and 446 nm upon excitation at 300 and 360 nm, respectively (Figure 7). The high-energy emission band is obviously due to intramolecular charge transfer in **1**, which is revealed from comparison of the absorption spectrum (Figure 6) and excitation spectrum at λ_em_ = 340 nm (Figure 7). This emission band is remarkably red-shifted (~100 nm) upon excitation at 360 nm. This large shift together with comparison of the absorption spectrum (Figure 6) and excitation spectrum at λ_em_ = 450 nm (Figure 7) has allowed to conclude that the low-energy emission is most likely due the origin of a new species upon excitation. Particularly, since the molecule of **1** is characterized by prominent intramolecular hydrogen bonding (Figure 4, Table 2), it might undergo a tautomeric transformation in the excited state, yielding a zwitterion structure 1*, formed upon transition of the phenolic OH hydrogen atom to the tertiary nitrogen atom of the 1,2,3,4-tetrahydropyrimidine fragment (Figure 8). The latter molecule might isomerize with the formation of a zwitterionic form of the corresponding Schiff base 1*′, which is, in turn, structurally related to the corresponding *cis*-keto form 1*″ (Figure 8) [58,59,60].

We have also applied the DFT calculations to reveal structural and electronic features of **1**, which structure was first optimized in gas phase. We have used the crystal structure geometry of Molecule A as a starting model for structural optimization. Notably, both enantiomeric forms of Molecule A yielded the same results of calculations and, for the sake of brevity, we focused on the *R*-isomer. The calculated geometrical parameters in the optimized structure of the *R*-isomer of **1** are in good agreement with the experimental ones (Table 1); however, a notable feature in the optimized structure of the *R*-isomer of **1** can be highlighted. Particularly, in the intramolecular hydrogen bonding O1–H1∙∙∙N1 the O1–H1 bond is 0.16 Å longer, while the H1∙∙∙N1 interaction is about 0.12 Å shorter in comparison to the experimental results (Table 2). Obviously, this discrepancy is explained by the DFT calculations performed in gas phase; thus, the optimized structure of **1** tends to adopt a zwitterionic isomeric form 1* (Figure 8).

**Figure 9:**
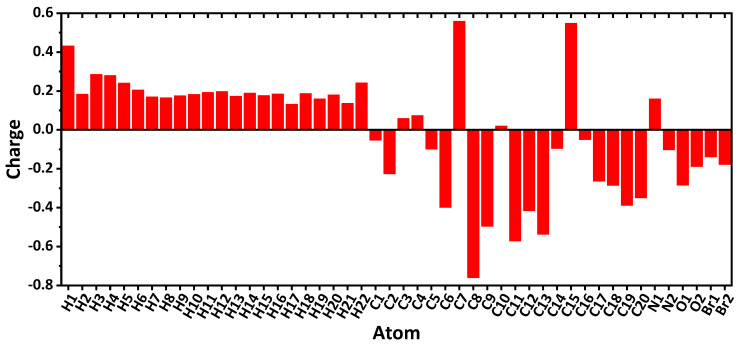
Mulliken atomic charges in the optimized structure of the *R*-isomer of **1**, obtained using the DFT/B3LYP/6-311++G(d,p) method (see Figure 4 for atoms labelling).

Analysis of the Mulliken atomic charges in the optimized structure of the *R*-isomer of **1** revealed that all the hydrogen atoms are positively charged with the highest value corresponding to the phenolic OH hydrogen atom, followed by H3, H4, H5, and H22 hydrogens (Figure 9). Of non-hydrogen atoms, the C7 and C15 are the most positively charged, followed by the N1 atom (Figure 9). The C8 carbon atom carries the most negative charge, followed by the C11, C13, and C9 atoms (Figure 9).

The calculated IR and ^1^H NMR of the optimized structure of the *R*-isomer of **1** do not contradict the experimental results and some discrepancies are due to optimization of the structure in gas phase (Figure 2 and Figure 3, Table 3). It should be noted that all the frequencies in the calculated IR spectrum were found to be positive, indicating local energy minima for the optimized structure.

The calculated UV-vis spectrum of the *R*-isomer of **1** in gas phase is in good agreement with the experimental one, and also exhibits bands exclusively in the UV region (Figure 6). Main transitions responsible for the bands in the calculated UV-vis spectrum are listed in Table 4.

According to the DFT calculations, the energies of the HOMO and LUMO of the *R*-isomer of **1** in gas phase are −6.02787 and −1.16302 eV, respectively, with the corresponding energy gap of 4.86485 eV (Table 5). The ionization potential (*I*) and the electron affinity (*A*) value are large (Table 5) indicating low electron-donating and high electron-accepting properties. Chemical potential (*μ*) is −3.59545 eV, indicating electron-accepting ability and the low donating ability, which is supported by the corresponding high value of electronegativity, *χ* (Table 5). The electrophilicity index (*ω*), which is denoted as the energy of stabilization to accept electrons, is 2.65727 eV, indicating the pronounced electrophilic nature. Finally, the calculated structure can accept about 1.5 electrons, as evidenced from the corresponding ΔN_max_ value (Table 5).

**Figure 10:**
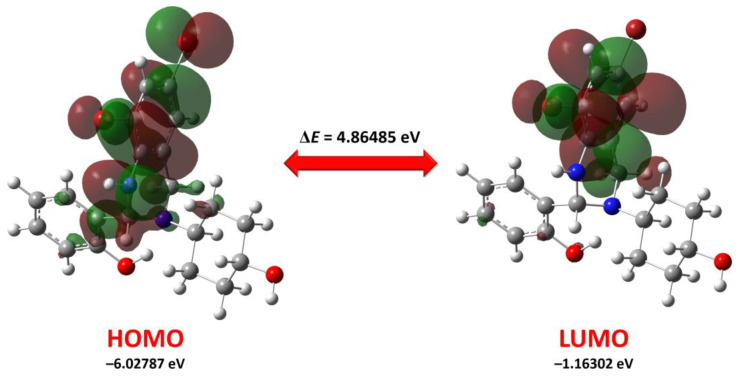
Energy levels and views on the electronic isosurfaces of the HOMO and LUMO of the optimized structures of the *R*-isomer of **1**, obtained using the DFT/B3LYP/6-311++G(d,p) method.

We have also visualized HOMO and LUMO for the *R*-isomer of **1**. It was found that the HOMO is mainly delocalized over the 6,8-dibromo-1,2,3,4-tetrahydroquinazoline fragment, while LUMO is mainly spread over the 6-bromophenylene fragment and the methylene group of the 1,2,3,4-tetrahydropyrimidine fragment (Figure 10).

**Figure 11:**
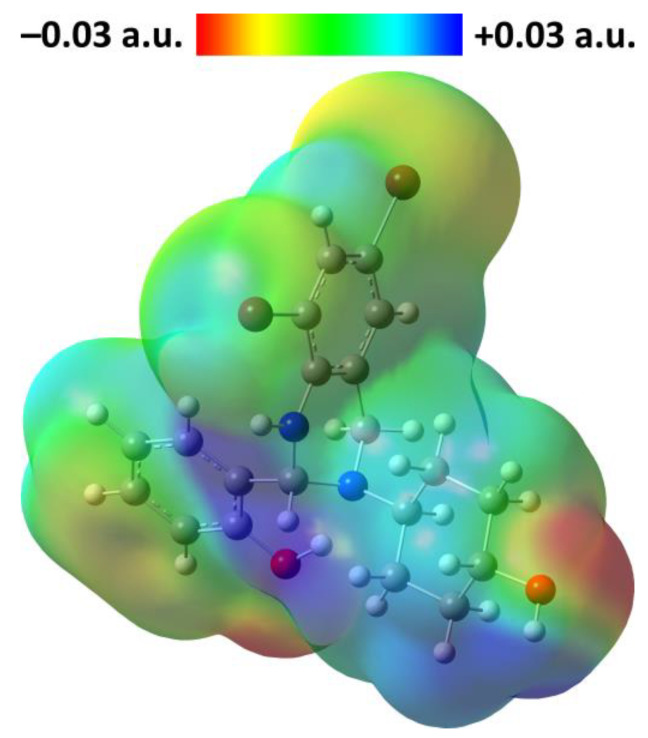
View of the molecular electrostatic potential surface of the optimized structure of the *R*-isomer of **1** in gas phase, obtained using the DFT/B3LYP/6-311++G(d,p) method.

The electrophilic and nucleophilic sites in the discussed optimized structure of the *R*-isomer of **1** were examined using the molecular electrostatic potential (MEP) analysis. The red and blue colours of the MEP surface correspond to electron-rich (nucleophilic) and electron-deficient (electrophilic) regions, respectively. On the MEP surface the most pronounced nucleophilic centers are located on the both hydroxyl oxygen atoms (Figure 11). As the most electrophilic region the cyclohexanol OH and NH hydrogen atoms, followed by H2, H7 and H14 hydrogens, can be highlighted (Figure 11).

**Figure 12:**
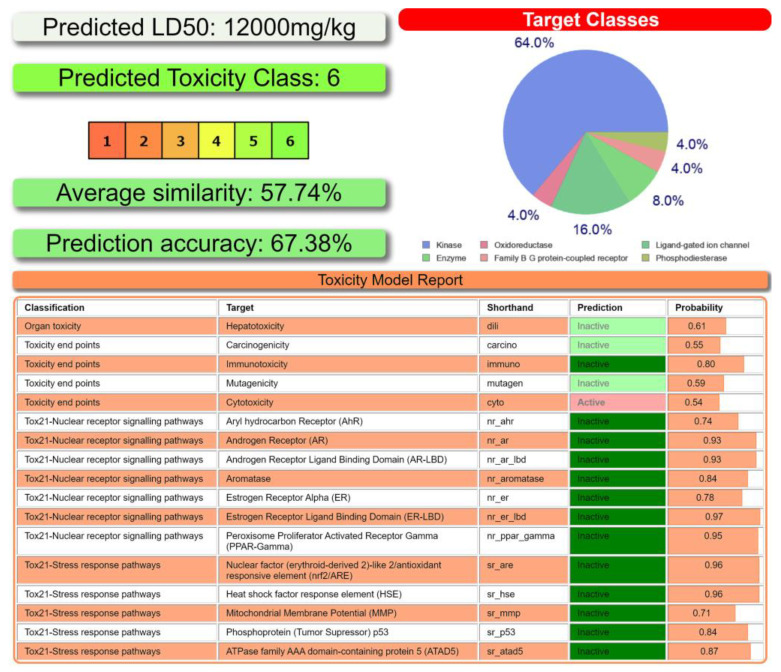
Toxicity results (**top left** and **bottom**) of the *R*-isomer of **1** calculated by ProTox-II. Druggability predictions (**top right**) of the *R*-isomer of **1** calculated by SwissADME.

According to ProTox-II, a virtual lab for the prediction of toxicities of small molecules [61,62], the *R*-isomer of **1** belongs to a sixth class of toxicity, and is likely a pronounced inhibitor of kinase, ligand-gated ion channel, enzyme, oxidoreductase, family B G protein-coupled receptor, and phosphodiesterase with the probabilities of 64.0%, 16.0%, 8.0%, 4.0%, 4.0%, and 4.0%, respectively (Figure 12). According to the Toxicity Model Report, the *R*-isomer of **1** was revealed to be cytotoxic (Figure 12).

**Figure 13:**
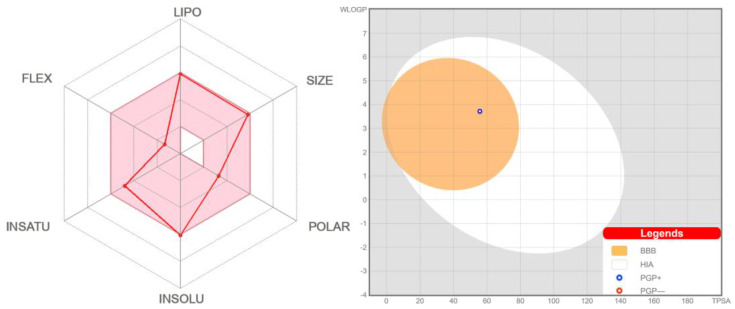
Bioavailability radar (**left**) for the *R*-isomer of **1** within the domain borders of ADME properties, calculated by SwissADME. The coloured zone of the radar is the suitable physicochemical space for oral bioavailability. BOILED-Egg model (**right**) of the *R*-isomer of **1** calculated by SwissADME.

As evidenced from the SwissADME [63] bioavailability radar, the discussed compound is preferred in all the six parameters, namely lipophilicity, size, polarity, insolubility, insaturation, and flexibility (Figure 13); thus, it is predicted to be suitable for oral bioavailability.

The BOILED-Egg method was found to be efficient to predict the human blood–brain barrier (BBB) penetration and gastrointestinal absorption [64]. This approach is based on lipophilicity (WLOGP) and polarity (topological polar surface area, TPSA) (Figure 13). Points located in the yellow region (BOILED-Egg’s yolk) are molecules predicted to passively permeate through the BBB, while points located in the white region (BOILED Egg’s white) are molecules predicted to be passively absorbed by the gastrointestinal tract. Blue (PGP+) and red (PGP−) dots are for molecules predicted to be effluated and not to be effluated from the central nervous system by the P-glycoprotein, respectively. As evidenced from the blue dot position for the *R*-isomer of **1**, both BBB penetration property and gastrointestinal absorption property are positive with the positive PGP effect on the molecule (Figure 13).

We have further applied a molecular docking approach for both *R*-isomer and *S*-isomer with a series of the SARS-CoV-2 proteins. Docking is the best option to diminish the time and cost of synthesis and to increase the influence of the medicines; in addition, it is considered as a current and advantageous method to have insight information of the possible binding site of the ligand in the protein. The target structures were primarily selected in accordance with the structural features of the virus [65,66] as well as based on biological mechanisms and functions that can be utilized to reduce, prevent, or treat the virus [67] (Table 6).

**Figure 14:**
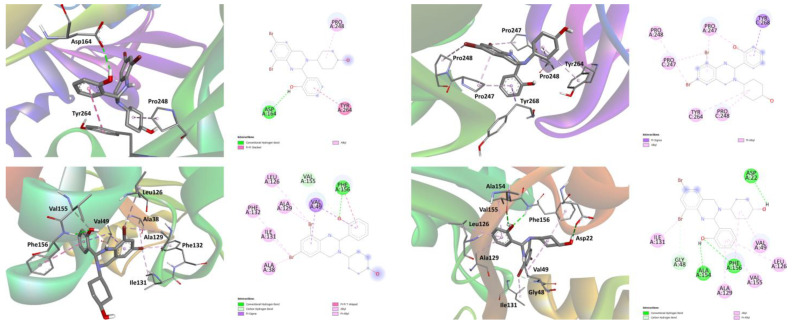
3D and 2D views on the interaction of the *R*-isomer (**left**) and *S*-isomer (**right**) of **1** with (from **top** to **bottom**) Papain-like protease (PLpro) and nonstructural protein 3 (Nsp3_range 207–379-AMP).

According to the docking analysis results, both isomers of **1** were found to be active against all the applied SARS-CoV-2 proteins with the best binding affinity with Papain-like protease (PLpro) and nonstructural protein 3 (Nsp3_range 207–379-AMP) (Figure 14, Table 6). Interactions responsible for binding of isomers of **1** and **2** with these two proteins are shown in Figure 14 and collected in Table 7.

The obtained molecular docking results for both isomers of **1** are comparable with those found for initial redocked ligands [44], Remdesivir [44], Molnupiravir [47], and different tautomers of salen [51] and betulin [53], and superior to those calculated for Favipiravir [44]; furthermore, both isomers of **1**, in general, interact with the applied SARS-CoV-2 proteins significantly more efficiently in comparison to the parent ambroxol [48]. Thus, **1** can be considered as a possible agent of further detailed investigation against COVID-19.

We have also revealed ligand efficiency scores shed more light on the bioactivity of both isomers of **1** towards the applied SARS-CoV-2 proteins. As such, for all complexes we have calculated inhibition constant (*K_i_*), miLogP, ligand efficiency (LE), ligand efficiency_scale (LE_Scale), fit quality (FQ), and ligand-efficiency-dependent lipophilicity (LELP) [68,69,70,71,72,73] (Table 6); furthermore, for comparison we have also calculated the same ligand efficiency scores for complexes of the studied proteins with initial ligands (Table 6). Notably, the *K_i_* value must be as low as possible for a more efficient inhibition and should fall in the μM range for a compound to be considered as a hit, and >10 nM for a drug [72]. Furthermore, for a compound to be considered as a hit, the LE, FQ, and LELP parameters are recommended as ≥0.3, ≥0.8 and from −10 to 10, respectively [72]. Of all the complexes of the applied proteins, the ligand efficiency scores for complexes with the both isomers of **1** with Papain-like protease (PLpro) as well as for the complex of the *S*-isomer with nonstructural protein 3 (Nsp3_range 207–379-AMP) are close to be within the recommended ranges for a hit, although the LELP values are somewhat out of the recommended range (Table 6).

**Figure 15:**
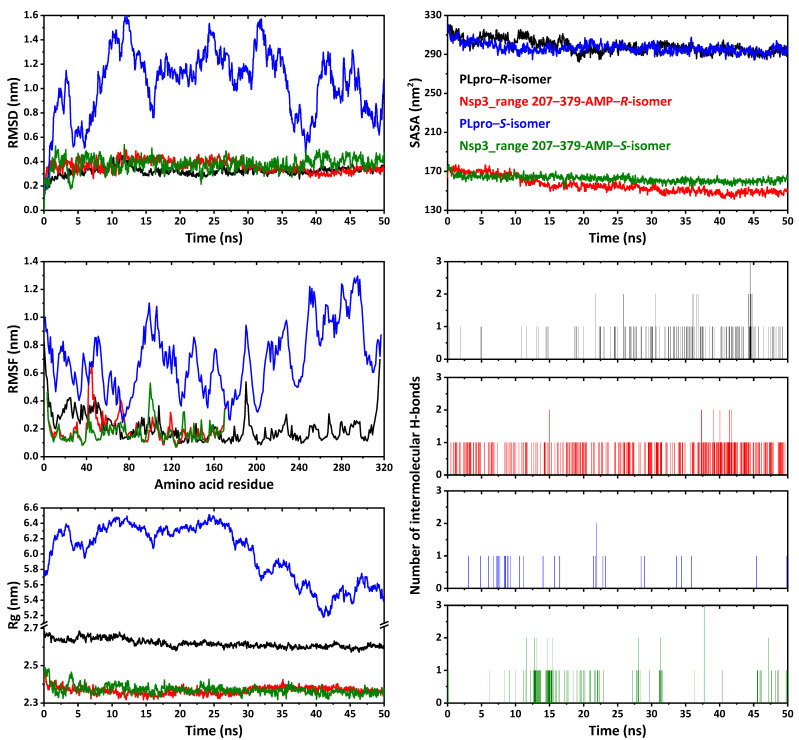
RMSD, RMSF, Rg, SASA, and intermolecular hydrogen bonds analysis profiles of complexes of the *R*-isomer and *S*-isomer of **1** with Papain-like protease (PLpro) and nonstructural protein 3 (Nsp3_range 207–379-AMP).

We have additionally performed molecular dynamics simulations of the 50 ns time to evaluate interactions in complexes PLpro–*R*/*S*-isomer and Nsp_range 207–379-AMP–*R*/*S*-isomer. Complexes PLpro–*R*-isomer and Nsp_range 207–379-AMP–*R*/*S*-isomer each showed a highly stable root mean square deviation (RMSD) over the whole simulation time with the average values of 0.322, 0.362, and 0.380 nm, reaching the maximum values of 0.467, 0.496, and 0.539 nm, respectively (Figure 15). Contrarily, complex PLpro–*S*-isomer showed much higher RMSD over the whole simulation time reaching the value of about 1.6 nm with the average value of 1.021 nm (Figure 15), indicating its pronounced instability. The root mean square fluctuation (RMSF) value for complexes PLpro–*R*-isomer and Nsp_range 207–379-AMP–*R*/*S*-isomer was below 0.829, 0.657, and 0.529 nm, respectively (Figure 15). The strongest fluctuations of amino acid residues for complexes PLpro–*R*-isomer and Nsp_range 207–379-AMP–*R*/*S*-isomer are listed in Table 8. The radius of gyration (Rg) values for complexes PLpro–*R*-isomer and Nsp_range 207–379-AMP–*R*/*S*-isomer form relatively stable profiles (Figure 15), with the values varying in the ranges 2.572–2.686, 2.317–2.475, and 2.318–2.488 nm, respectively. The solvent accessible surface area (SASA) profiles were calculated for predicting the interaction between complexes and solvents. It was also established that the binding of the *R*-isomers to PLpro and Nsp3_range 207–379-AMP, and of the *S*-isomer to Nsp3_range 207–379-AMP did not impair the proteins’ interaction with the solvent molecule and the stability of the proteins (Figure 15). During the 50 ns simulation time, the average SASA was calculated as 298.41, 155.66, and 162.22 nm^2^ for complexes PLpro–*R*-isomer and Nsp_range 207–379-AMP–*R*/*S*-isomer, respectively. It was also established that in complex Nsp_range 207–379-AMP–*R*-isomer mainly 1 intermolecular hydrogen bond is formed during almost the whole simulation time, while in complex PLpro–*R*-isomer also 1 intermolecular hydrogen bond is formed but at about 18–50 ns (Figure 15). Complex Nsp_range 207–379-AMP–*S*-isomer is also characterized by 1 intermolecular hydrogen bond at about 8–23, 27–32, and 46–48 ns (Figure 15).

## 3. Materials and Methods

### 3.1. Physical Measurements

The IR spectrum in a KBr pellet was recorded with a FT-IR FSM 1201 spectrometer in the range 400–4000 cm^−1^. The ^1^H NMR spectrum in DMSO-*d*_6_ were obtained on a Bruker Avance II 400 MHz spectrometer at 25 °C. Chemical shifts are reported with reference to SiMe_4_. UV–vis and fluorescent spectra from the 10^−4^ M freshly prepared solutions in freshly distilled MeOH were recorded on an Agilent 8453 instrument and RF-5301PC Shimadzu spectrofluorimeter, respectively. Powder X-ray diffraction was carried out using a Rigaku Ultima IV X-ray powder diffractometer. The parallel beam mode was used to collect the data (λ = 1.54184 Å). Elemental analyses were performed on a Thermo Flash 2000 CHNS analyzer (Waltham, MA, USA).

### 3.2. Synthesis

A solution of salicylaldehyde (0.6 mmol, 0.073 g) in ethanol (10 mL) was added to a solution of ambroxol hydrochloride (0.5 mmol, 0.207 g) and KOH (0.5 mmol, 0.028 g) in the same solvent (20 mL). The mixture was heated at reflux for about 2 h. The resulting hot solution was filtered and allowed to cool to room temperature to give colorless crystals **1**∙0.5EtOH suitable for single-crystal X-ray diffraction. Yield: 0.217 g (86%). Anal. Calc. for C_21_H_25_Br_2_N_2_O_2.5_ (505.25): C 49.92, H 4.99, and N 5.54; found: C 50.04, H 5.07, and N 5.48%.

### 3.3. Single-Crystal X-ray Diffraction

The X-ray diffraction data for **1**∙0.5EtOH were collected at 150(2) K on a Bruker Smart Apex-II diffractometer, equipped with a CCD detector (Mo-Kα, λ = 0.71073 Å, graphite monochromator). Semi-empirical absorption correction was applied by the SADABS program [74]. The structure was solved by direct methods and refined by the full-matrix least squares in the anisotropic approximation for non-hydrogen atoms. The calculations were carried out by the SHELX-2014 program package [75] using Olex2 1.2 [76]. CCDC 2235606 contains the supplementary crystallographic data. These data can be obtained free of charge via https://www.ccdc.cam.ac.uk/structures or from the Cambridge Crystallographic Data Centre, 12 Union Road, Cambridge CB2 1EZ, UK; fax: (+44)-1223-336-033; or e-mail: deposit@ccdc.cam.ac.uk. 2(C_20_H_22_Br_2_N_2_O_2_), C_2_H_6_O; *Mr* = 1010.50 g mol^−1^, triclinic, space group *P*–1, *a* = 5.7020(5), *b* = 16.8765(16), *c* = 21.6839(19) Å, *α* = 93.483(3), *β* = 97.142(3), *γ* = 98.957(3)°, *V* = 2038.4(3) Å^3^, *Z* = 2, *ρ* = 1.646 g cm^−3^, *μ*(Mo-Kα) = 3.999 mm^−1^, reflections: 18936 collected, 7941 unique, *R*_int_ = 0.054, *R*_1_(all) = 0.0737, *wR*_2_(all) = 0.1105, *S* = 1.027.

### 3.4. DFT Calculations

The crystal structure geometries of the *R*-isomer and *S*-isomer of **1** were used as starting models for structural optimization. The ground state geometries were fully optimized without symmetry restrictions. The calculations were performed by means of the GaussView 6.0 molecular visualization program [77] and Gaussian 09, Revision D.01 program package [78] using the density functional theory (DFT) method with Becke-3-parametre-Lee–Yang–Parr (B3LYP) hybrid functional [79,80] and 6-311++G(d,p) [79,81] basis set. The vibration frequencies were calculated for the optimized structure in gas phase and no imaginary frequencies were obtained. The electronic isosurfaces of the HOMO and LUMO orbitals and MEP surfaces were generated from the fully optimized ground state geometry obtained using the B3LYP/6-311++G(d,p) method. The absorption and ^1^H NMR spectra of the fully optimized ground state geometry were simulated at the TD-DFT/B3LYP/6-311++G(d,p) and GIAO/B3LYP/6-311++G(2d,p) levels, respectively.

### 3.5. Molecular Docking

Molecular docking of both isomers of **1** with a series of the SARS-CoV-2 proteins were carried using the CB-Dock2 server [82,83], which reveals protein cavities to guide blind docking by the algorithm of AutoDock Vina [84]. The targeted protein structures were subtracted from the RCSB PDB database [85] and were pretreated before the docking, including water removing and inserting hydrogen atoms and missing residues and charges. Docking results were visualized in BIOVIA Discovery Studio 2020 [86].

### 3.6. Molecular Dynamics Simulation

Molecular dynamics simulations were performed using the WebGro on-line service [87]. Parameters such as root mean square deviation (RMSD), root mean square fluctuation (RMSF), radius of gyration (Rg), solvent accessible surface area (SASA), and intermolecular hydrogen bonds were assessed. The complex was prepared for molecular dynamics simulations using GROMOS96 54a7 forcefield and was equilibrated using the canonical (NVT) and the isothermal-isobaric (NPT) ensembles. The ligand topology was generated with the PRODRG tool [88]. Simple point charge (SPC) was used as a solvent model (triclinic water box with size 50 × 75 × 70 Å) for protein–ligand complex [89]. This system was neutralized by adding sodium or chlorine ions based on the total charges. For minimization of the system before molecular dynamics simulations the steepest descent algorithm (5000 steps) was applied. The simulations were performed in the presence 0.15 M NaCl using the constant temperature (310 K) and pressure (1.0 bar). Approximate number of frames per simulation was 1000. The simulation time was set to 50 ns.

### 3.7. In Silico Drug-Likeness Analysis

Bioavailability, druggability, as well as absorption, distribution, metabolism, excretion, and toxicity properties were evaluated using the SwissADME [63], BOILED-Egg [64], and ProTox-II [61,62] tools.

## 4. Conclusions

We have synthesized a novel 1,2,3,4-tetrahydroquinazoline derivative, named 2-(6,8-dibromo-3-(4-hydroxycyclohexyl)-1,2,3,4-tetrahydroquinazolin-2-yl)phenol (**1**), which was obtained from the hydrochloride of 4-((2-amino-3,5-dibromobenzyl)amino)cyclohexan-1-ol (ambroxol hydrochloride) and salicylaldehyde in EtOH. The resulting compound was produced in the form of colorless crystals of the composition **1**∙0.5EtOH. The molecule of **1** contains a chiral tertiary carbon of the 1,2,3,4-tetrahydropyrimidine fragment and the crystal structure of **1**∙0.5EtOH is a racemate. It was established that the compound absorbs in MeOH exclusively in the UV region up to about 350 nm; furthermore, **1**∙0.5EtOH in the same solvent exhibits dual emission and the spectra contains bands at about 340 and 446 nm. While the high-energy emission band is due to intramolecular charge transfer, the low-energy emission is most likely due the excitation induced origin of a new species of **1**, formed upon transition of the phenolic OH hydrogen atom to the tertiary nitrogen atom of the 1,2,3,4-tetrahydropyrimidine fragment.

The DFT based calculations allowed to establish values of the global chemical reactivity descriptors, which revealed electron accepting and donating abilities of the reported compound, as well as its molecular electrostatic potential surface, which revealed electrophilic and nucleophilic sites.

ADMET properties of the *R*-isomer of **1** were evaluated using the SwissADME, BOILED-Egg and ProTox-II tools, which predicted its positive human blood–brain barrier penetration and gastrointestinal absorption properties with the positive PGP effect on the molecule. According to the molecular docking analysis, both isomers of **1** were found to be active against all the applied SARS-CoV-2 proteins with the best binding affinity with Papain-like protease (PLpro) and nonstructural protein 3 (Nsp3_range 207–379-AMP). Ligand efficiency scores for both isomers of **1** inside the binding sites of the applied proteins were also revealed and compared with the initial ligands. Of all the complexes, the ligand efficiency scores for complexes of the both isomers of **1** with Papain-like protease (PLpro) as well as for the complex of the *S*-isomer with nonstructural protein 3 (Nsp3_range 207–379-AMP) are close to be within the recommended ranges for a hit, although the LELP values are somewhat out of the recommended range. Finally, molecular dynamics simulations of the 50 ns time revealed that complexes of the *R*-isomer with Papain-like protease (PLpro) and nonstructural protein 3 (Nsp3_range 207–379-AMP), and the complex of the *S*-isomer with nonstructural protein 3 (Nsp3_range 207–379-AMP) are stable, while the complex of the *S*-isomer with Papain-like protease (PLpro) was found to be highly unstable.

## Figures and Tables

**Table 1:** Selected bond lengths (Å), and bond and dihedral angles (°) in Molecule A and Molecule B in the crystal structure of **1**∙0.5EtOH, and optimized structure of the *R*-isomer of **1** in gas phase, obtained using the DFT/B3LYP/6-311++G(d,p) method.

	Molecule A	Molecule B	Optimized Structure
Bond length			
N1–C1	1.481(6)	1.476(6)	1.471
N1–C8	1.482(6)	1.481(7)	1.474
N1–C9	1.487(6)	1.405(12), 1.593(12)	1.492
N2–C1	1.453(6)	1.452(6)	1.460
N2–C2	1.382(6)	1.393(6)	1.382
Br1–C3	1.895(5)	1.897(5)	1.923
Br2–C5	1.900(5)	1.906(5)	1.918
O1–C16	1.376(5)	1.372(6)	1.361
O2–C12	1.434(6)	1.431(6)	1.431
C1–C15	1.537(6)	1.512(6)	1.540
C2–C3	1.409(6)	1.396(7)	1.405
C2–C7	1.401(7)	1.409(6)	1.411
C3–C4	1.380(7)	1.384(7)	1.389
C4–C5	1.385(7)	1.377(7)	1.390
C5–C6	1.378(6)	1.377(7)	1.391
C6–C7	1.391(7)	1.378(7)	1.391
C7–C8	1.511(6)	1.502(7)	1.521
C9–C10	1.518(7)	1.498(14), 1.516(14)	1.543
C9–C14	1.526(7)	1.245(13), 1.765(15)	1.537
C10–C11	1.532(6)	1.538(12), 1.545(13)	1.537
C11–C12	1.513(7)	1.614(10), 1.431(11)	1.525
C12–C13	1.506(7)	1.480(11), 1.460(11)	1.532
C13–C14	1.530(6)	1.497(15), 1.534(14)	1.537
C15–C16	1.401(7)	1.418(7)	1.412
C15–C20	1.386(6)	1.389(7)	1.397
C16–C17	1.387(7)	1.382(7)	1.397
C17–C18	1.384(6)	1.372(7)	1.390
C18–C19	1.378(7)	1.396(7)	1.394
C19–C20	1.396(7)	1.392(7)	1.393
Bond angle			
N1–C1–N2	111.1(4)	110.5(4)	110.6
N1–C1–C15	109.7(4)	110.4(4)	110.7
N2–C1–C15	113.7(4)	114.5(4)	114.2
N1–C8–C7	115.6(4)	114.1(4)	114.3
N2–C2–C7	119.5(4)	119.1(4)	119.6
C1–N1–C8	110.5(3)	108.8(4)	110.2
C1–N1–C9	117.0(3)	112.0(6), 114.9(5)	118.4
C1–N2–C2	118.9(4)	119.1(4)	119.5
C8–N1–C9	114.3(3)	124.4(5), 108.4(5)	114.8
C2–C7–C8	119.3(4)	119.2(4)	118.9
Dihedral angle ^1^			
N1–C1–N2–C2	48.8(5)	−46.3(5)	45.7
N2–C1–N1–C8	−55.5(5)	60.3(5)	−58.6
N1–C8–C7–C2	−17.5(6)	22.4(6)	−19.2
N2–C2–C7–C8	7.9(6)	−5.7(6)	4.0
C1–N1–C8–C7	41.0(5)	−49.0(5)	46.2
C1–N2–C2–C7	−24.2(6)	18.1(6)	−17.9
N1–C1–C15–C16	42.3(6)	−44.8(6)	45.6
N1–C1–C15–C20	−142.4(4)	138.0(5)	−139.2
N2–C1–C15–C16	167.3(4)	−170.2(4)	171.2
N2–C1–C15–C20	−17.4(7)	12.5(6)	−13.6
C1–N1–C9–C10	−93.5(4)	90.9(9), 133.6	−68.2
C1–N1–C9–C14	62.3(5)	−110.6(13), −135.1	60.3
C8–N1–C9–C10	66.8(5)	−43.2(11), 11.8	64.7
C8–N1–C9–C14	−166.4(4)	115.2(12), 103.0	−166.8

^1^ Dihedral angles must be compared by their absolute values.

**Table 2:** Intramolecular hydrogen bond lengths (Å) and angles (°) in Molecule A, Molecule B, and optimized structure of the *R*-isomer of **1** in gas phase, obtained using the DFT/B3LYP/6-311++G(d,p) method.

	D–X∙∙∙A	*d*(D–X)	*d*(X∙∙∙A)	*d*(D∙∙∙A)	∠(DXA)
Molecule A	O1–H1∙∙∙N1	0.82	1.94	2.659(5)	146
Molecule B		0.82	1.95	2.668(6)	146
Optimized structure		0.98	1.82	2.713	149

**Table 3:** Signals for the calculated ^1^H NMR spectrum of the optimized structure of the *R*-isomer of **1** in gas phase, obtained using the DFT/GIAO/B3LYP/6-311++G(2d,p) method (see Figure 3 for atoms labelling).

δ_H_ (ppm)	Hydrogen	δ_H_ (ppm)	Hydrogen	δ_H_ (ppm)	Hydrogen
0.85	H22	1.96	H10	5.58	H2
1.11	H11	2.97	H8	6.99	H5 + H18 + H20
1.37	H14	3.47	H7	7.26	H21
1.62	H9 + H12	3.69	H13	7.42	H4
1.76	H16	4.02	H6	7.47	H19
1.86	H15 + H17	5.11	H3	10.07	H1

**Table 4:** Selected values of the calculated UV-vis spectrum (Figure 6) for the optimized structure of the *R*-isomer of **1** in gas phase, obtained using the TD-DFT/B3LYP/6-311++G(d,p) method.

λ_max_ (nm)	Oscillator Strength	Transition
295.73	0.0526	HOMO → LUMO (92.5%)
255.18	0.0688	HOMO−1 → LUMO+2 (46.3%)
		HOMO−1 → LUMO+3 (24.2%)
252.09	0.1702	HOMO → LUMO+2 (27.6%)
		HOMO → LUMO+3 (55.3%)
214.80	0.3098	HOMO−4 → LUMO (63.7%)
211.04	0.0809	HOMO−3 → LUMO+2 (50.5%)
		HOMO−3 → LUMO+3 (23.0%)
209.01	0.979	HOMO−4 → LUMO+2 (35.3%)
		HOMO−3 → LUMO+3 (10.6%)
		HOMO → LUMO+13 (12.3%)
192.92	0.0714	HOMO−3 → LUMO+4 (8.4%)
		HOMO−3 → LUMO+5 (8.9%)
		HOMO−3 → LUMO+8 (8.4%)
		HOMO−3 → LUMO+10 (21.6%)
		HOMO−2 → LUMO+9 (8.1%)
189.56	0.0645	HOMO−4 → LUMO+7 (35.0%)
		HOMO−3 → LUMO+8 (12.3%)
		HOMO → LUMO+18 (8.7%)
189.49	0.0405	HOMO−6 → LUMO+2 (42.9%)
		HOMO−6 → LUMO+3 (8.8%)
		HOMO−3 → LUMO+8 (8.8%)
		HOMO → LUMO+18 (10.3%)
187.97	0.0700	HOMO−3 → LUMO+7 (9.5%)
		HOMO → LUMO+19 (52.0%)
187.75	0.2730	HOMO−3 → LUMO+7 (36.2%)
		HOMO → LUMO+19 (11.3%)
		HOMO → LUMO+21 (10.2%)
186.81	0.0410	HOMO−3 → LUMO+8 (32.3%)
		HOMO−3 → LUMO+10 (29.3%)
183.82	0.0536	HOMO−3 → LUMO+9 (16.6%)
		HOMO−1 → LUMO+17 (21.8%)
		HOMO−1 → LUMO+20 (14.1%)
179.97	0.1364	HOMO−10→ LUMO (11.4%)
		HOMO−9 → LUMO (56.4%)

**Table 5:** Frontier molecular HOMO and LUMO orbitals, gap value, and descriptors for the optimized structures of the *R*-isomer of **1** in gas phase, obtained using the DFT/B3LYP/6-311++G(d,p) method.

*E*_HOMO_ (eV)	−6.02787
*E*_LUMO_ (eV)	−1.16302
Δ*E*_LUMO−HOMO_ = *E*_LUMO_ − *E*_HOMO_ (eV)	4.86485
Ionization energy, *I* = −*E*_HOMO_ (eV)	6.02787
Electron affinity, *A* = −*E*_LUMO_ (eV)	1.16302
Electronegativity, *χ* = (*I* + *A*)/2 (eV)	3.59545
Chemical potential, *μ* = −*χ* (eV)	−3.59545
Global chemical hardness, *η* = (*I* − *A*)/2 (eV)	2.43243
Global chemical softness, *S* = 1/(2*η*) (eV^−1^)	0.20556
Global electrophilicity index, *ω* = *μ*^2^/(2*η*) (eV)	2.65727
Maximum additional electric charge, ΔN_max_ = −*μ*/*ƞ*	1.47813

**Table 6:** Ligand efficiency scores for the initial ligands, and *R*-isomer and *S*-isomer of **1** inside the binding sites of the listed proteins.

Ligand Efficiency Score	Initial Ligand *	*R*-isomer	*S*-isomer
Main protease (Mpro) (PDB code 6LU7) Docking center (*x*, *y*, *z*) = −12, 13, 69; Docking size (*x*, *y*, *z*) = 21, 21, 21; Cavity volume = 448 Å^3^			
Binding energy (BE, kcal/mol)	−7.4(1)	−7.2(0)	−7.6(0)
Inhibition constant (*K_i_* = *e*^(−BE/*RT*)^, μM) **	3.76	5.28	2.69
miLogP	2.32	4.73	
Ligand efficiency (LE = −BE/(Heavy atoms), kcal/(mol HA)	0.151	0.277	0.292
LE_Scale (0.0715 + 7.5328/(HA) + 25.7079/(HA^2^) − 361.4722/(HA^3^))	0.233	0.379	
Fit quality (FQ = LE/LE_Scale)	0.649	0.731	0.772
Ligand-efficiency-dependent lipophilicity (LELP = miLogP/LE)	15.362	17.081	16.182
Papain-like protease (PLpro) (PDB code 6WUU) Docking center (*x*, *y*, *z*) = 25, 68, −2; Docking size (*x*, *y*, *z*) = 35, 35, 35; Cavity volume = 8091 Å^3^			
Binding energy (BE, kcal/mol)	−8.6(1)	−8.3(0)	−8.2(0)
Inhibition constant (*K_i_* = *e*^(−BE/*RT*)^, μM) **	0.50	0.82	0.98
miLogP	−1.61	4.73	
Ligand efficiency (LE = −BE/(Heavy atoms), kcal/(mol HA)	0.239	0.319	0.315
LE_Scale (0.0715 + 7.5328/(HA) + 25.7079/(HA^2^) − 361.4722/(HA^3^))	0.293	0.379	
Fit quality (FQ = LE/LE_Scale)	0.816	0.843	0.833
Ligand-efficiency-dependent lipophilicity (LELP = miLogP/LE)	−6.740	14.817	14.998
Nonstructural protein 3 (Nsp3_range 207–379-AMP) (PDB code 6W6Y) Docking center (*x*, *y*, *z*) = 9, −9, 11; Docking size (*x*, *y*, *z*) = 21, 21, 27; Cavity volume = 518 Å^3^			
Binding energy (BE, kcal/mol)	−7.2(0)	−7.7(0)	−8.3(0)
Inhibition constant (*K_i_* = *e*^(−BE/*RT*)^, μM) **	5.28	2.27	0.82
miLogP	−1.52	4.73	
Ligand efficiency (LE = −BE/(Heavy atoms), kcal/(mol HA)	0.313	0.296	0.319
LE_Scale (0.0715 + 7.5328/(HA) + 25.7079/(HA^2^) − 361.4722/(HA^3^))	0.418	0.379	
Fit quality (FQ = LE/LE_Scale)	0.749	0.782	0.843
Ligand-efficiency-dependent lipophilicity (LELP = miLogP/LE)	−4.856	15.971	14.817
Nonstructural protein 3 (Nsp3_range 207–379-MES) (PDB code 6W6Y) Docking center (*x*, *y*, *z*) = 24, 7, 50; Docking size (*x*, *y*, *z*) = 21, 21, 21; Cavity volume = 582 Å^3^			
Binding energy (BE, kcal/mol)	−5.8(0)	−7.7(1)	−7.9(0)
Inhibition constant (*K_i_* = *e*^(−BE/*RT*)^, μM) **	56.05	2.27	1.62
miLogP	−4.08	4.73	
Ligand efficiency (LE = −BE/(Heavy atoms), kcal/(mol HA)	0.483	0.296	0.304
LE_Scale (0.0715 + 7.5328/(HA) + 25.7079/(HA^2^) − 361.4722/(HA^3^))	0.669	0.379	
Fit quality (FQ = LE/LE_Scale)	0.723	0.782	0.802
Ligand-efficiency-dependent lipophilicity (LELP = miLogP/LE)	−8.441	15.971	15.567
RdRp-RNA (PDB code 7BV2) Docking center (*x*, *y*, *z*) = 100, 96, 104; Docking size (*x*, *y*, *z*) = 21, 21, 21; Cavity volume = 790 Å^3^			
Binding energy (BE, kcal/mol)	−6.6(0)	−7.7(0)	−7.2(0)
Inhibition constant (*K_i_* = *e*^(−BE/*RT*)^, μM) **	14.53	2.27	5.28
miLogP	−1.55	4.73	
Ligand efficiency (LE = −BE/(Heavy atoms), kcal/(mol HA)	0.264	0.296	0.277
LE_Scale (0.0715 + 7.5328/(HA) + 25.7079/(HA^2^) − 361.4722/(HA^3^))	0.391	0.379	
Fit quality (FQ = LE/LE_Scale)	0.676	0.782	0.731
Ligand-efficiency-dependent lipophilicity (LELP = miLogP/LE)	5.871	15.971	17.081
Nonstructural protein 14 (N7-MTase) (PDB code 5C8S) Docking center (*x*, *y*, *z*) = 4, −31, 13; Docking size (*x*, *y*, *z*) = 32, 21, 33; Cavity volume = 4987 Å^3^			
Binding energy (BE, kcal/mol)	−10.7(0)	−7.6(1)	−7.5(0)
Inhibition constant (*K_i_* = *e*^(−BE/*RT*)^, μM) **	0.01	2.96	3.18
miLogP	−4.67	4.73	
Ligand efficiency (LE = −BE/(Heavy atoms), kcal/(mol HA)	0.214	0.292	0.288
LE_Scale (0.0715 + 7.5328/(HA) + 25.7079/(HA^2^) − 361.4722/(HA^3^))	0.230	0.379	
Fit quality (FQ = LE/LE_Scale)	0.932	0.772	0.762
Ligand-efficiency-dependent lipophilicity (LELP = miLogP/LE)	−21.822	16.182	16.397
Nonstructural protein 15 (endoribonuclease) (PDB code 6WLC) Docking center (*x*, *y*, *z*) = 74, −26, −29; Docking size (*x*, *y*, *z*) = 21, 21, 21; Cavity volume = 952 Å^3^			
Binding energy (BE, kcal/mol)	−7.5(1)	−6.4(1)	−5.3(1)
Inhibition constant (*K_i_* = *e*^(−BE/*RT*)^, μM) **	3.18	20.36	130.34
miLogP	−2.76	4.73	
Ligand efficiency (LE = −BE/(Heavy atoms), kcal/(mol HA)	0.357	0.246	0.204
LE_Scale (0.0715 + 7.5328/(HA) + 25.7079/(HA^2^) − 361.4722/(HA^3^))	0.449	0.379	
Fit quality (FQ = LE/LE_Scale)	0.795	0.650	0.538
Ligand-efficiency-dependent lipophilicity (LELP = miLogP/LE)	−7.728	19.216	23.204
Nonstructural protein 16 (GTA site) (PDB code 6WVN) Docking center (*x*, *y*, *z*) = 87, 15, 28; Docking size (*x*, *y*, *z*) = 21, 21, 21; Cavity volume = 700 Å^3^			
Binding energy (BE, kcal/mol)	−8.7(1)	−7.6(1)	−7.1(1)
Inhibition constant (*K_i_* = *e*^(−BE/*RT*)^, μM) **	0.42	2.69	6.25
miLogP	−5.69	4.73	
Ligand efficiency (LE = −BE/(Heavy atoms), kcal/(mol HA)	0.171	0.292	0.273
LE_Scale (0.0715 + 7.5328/(HA) + 25.7079/(HA^2^) − 361.4722/(HA^3^))	0.226	0.379	
Fit quality (FQ = LE/LE_Scale)	0.754	0.772	0.721
Ligand-efficiency-dependent lipophilicity (LELP = miLogP/LE)	−33.355	16.182	17.321
Nonstructural protein 16 (MGP site) (PDB code 6WVN) Docking center (*x*, *y*, *z*) = 105, 34, 29; Docking size (*x*, *y*, *z*) = 21, 21, 21; Cavity volume = 414 Å^3^			
Binding energy (BE, kcal/mol)	−6.7(0)	−6.7(1)	−6.6(0)
Inhibition constant (*K_i_* = *e*^(−BE/*RT*)^, μM) **	12.27	12.27	14.53
miLogP	−4.22	4.73	
Ligand efficiency (LE = −BE/(Heavy atoms), kcal/(mol HA)	0.203	0.258	0.254
LE_Scale (0.0715 + 7.5328/(HA) + 25.7079/(HA^2^) − 361.4722/(HA^3^))	0.313	0.379	
Fit quality (FQ = LE/LE_Scale)	0.648	0.680	0.670
Ligand-efficiency-dependent lipophilicity (LELP = miLogP/LE)	−20.785	18.355	18.633
Nonstructural protein 16 (SAM site) (PDB code 6WVN) Docking center (*x*, *y*, *z*) = 81, 27, 37; Docking size (*x*, *y*, *z*) = 21, 21, 21; Cavity volume = 655 Å^3^			
Binding energy (BE, kcal/mol)	−7.3(1)	−7.3(0)	−7.6(1)
Inhibition constant (*K_i_* = *e*^(−BE/*RT*)^, μM) **	4.46	4.46	2.69
miLogP	−5.01	4.73	
Ligand efficiency (LE = −BE/(Heavy atoms), kcal/(mol HA)	0.270	0.281	0.292
LE_Scale (0.0715 + 7.5328/(HA) + 25.7079/(HA^2^) − 361.4722/(HA^3^))	0.367	0.379	
Fit quality (FQ = LE/LE_Scale)	0.736	0.741	0.772
Ligand-efficiency-dependent lipophilicity (LELP = miLogP/LE)	−18.530	16.847	16.182

* (from top to bottom) Initial ligand = *N*-[(5-methylisoxazol-3-yl)carbonyl]alanyl-*L*-valyl-*N*~1~-((1*R*,2*Z*)-4-(benzyloxy)-4-oxo-1-{[(3*R*)-2-oxopyrrolidin-3-yl]methyl}but-2-enyl)-*L*-leucinamide; methyl 4-[2-[[(2~{*S*})-2-[[(2~{*S*})-2-acetamido-4-(1,3-benzothiazol-2-yl)butanoyl]amino]-3-azanyl-propanoyl]amino]ethanoylamino]butanoate; adenosine monophosphate; 2-morpholin-4-ium-4-ylethanesulfonate; [(2~{*R*},3~{*S*},4~{*R*},5~{*R*})-5-(4-azanylpyrrolo [2,1-f][1,2,4]triazin-7-yl)-5-cyano-3,4-*bis*(oxidanyl)oxolan-2-yl]methyl dihydrogen phosphate; [(2R,3S,4R,5R)-5-(2-amino-6-oxo-1H-purin-9-yl)-3,4-dihydroxy-oxolan-2-yl]methyl [[[(2R,3S,4R,5R)-5-(6-aminopurin-9-yl)-3,4-dihydroxy-oxolan-2-yl]methoxy-hydroxy-phosphoryl]oxy-hydroxy-phosphoryl] hydrogen phosphate; uridine-5′-monophosphate; [(2R,3S,4R,5R)-5-(2-amino-7-methyl-6-oxo-1H-purin-7-ium-9-yl)-3,4-dihydroxy-oxolan-2-yl]methyl [[[(2R,3S,4R,5R)-5-(6-aminopurin-9-yl)-3,4-dihydroxy-oxolan-2-yl]methoxy-hydroxy-phosphoryl]oxy-hydroxy-phosphoryl] hydrogen phosphate; 7-methyl-guanosine-5′-triphosphate; *S*-adenosylmethionine. ** *R* = 1.9872 × 10^−3^ kcal/(mol K), *T* = 298.15 K.

**Table 7:** The best types of interactions and distances of complexes of the *R*-isomer and *S*-isomer of **1** with Papain-like protease (PLpro) and nonstructural protein 3 (Nsp3_range 207–379-AMP).

Interaction	Distance (Å)	Bonding	Bonding Type
Papain-like protease (PLpro)–*R*-isomer			
A:*R*:H1A—A:ASP164:OD1	2.40881	Hydrogen Bond	Conventional Hydrogen Bond
A:TYR264—A:*R*	4.48693	Hydrophobic	π∙∙∙π Stacked
A:PRO248—A:*R*	4.17917	Hydrophobic	Alkyl
Nonstructural protein 3 (Nsp3_range 207–379-AMP)–*R*-isomer			
A:PHE156:HN—A:*R*:O1A	2.21013	Hydrogen Bond	Conventional Hydrogen Bond
A:VAL155:CA—A:*R*:O1A	3.41791	Hydrogen Bond	Carbon Hydrogen Bond
A:VAL49:CG2—A:*R*	3.71739	Hydrophobic	π∙∙∙Sigma
A:VAL49:CG2—A:*R*	3.79683	Hydrophobic	π∙∙∙Sigma
A:PHE156—A:*R*	5.59477	Hydrophobic	π∙∙∙π T–shaped
A:ALA38—A:*R*:Br2A	4.05446	Hydrophobic	Alkyl
A:ALA129—A:*R*:Br1A	4.02535	Hydrophobic	Alkyl
A:*R*:Br1A—A:LEU126	4.66976	Hydrophobic	Alkyl
A:*R*:Br2A—A:ILE131	4.55532	Hydrophobic	Alkyl
A:PHE132—A:*R*:Br2A	4.62370	Hydrophobic	π∙∙∙Alkyl
Papain-like protease (PLpro)–*S*-isomer			
C:TYR268:CB—A:*S*	3.91233	Hydrophobic	π∙∙∙Sigma
C:PRO248—A:*S*	4.48460	Hydrophobic	Alkyl
A:*S*:Br1A—C:PRO247	4.27482	Hydrophobic	Alkyl
A:*S*:Br2A—A:PRO248	5.39502	Hydrophobic	Alkyl
C:TYR264—A:*S*	5.36252	Hydrophobic	π∙∙∙Alkyl
A:*S*—A:PRO247	4.04681	Hydrophobic	π∙∙∙Alkyl
A:*S*—A:PRO247	4.67897	Hydrophobic	π∙∙∙Alkyl
Nonstructural protein 3 (Nsp3_range 207–379-AMP)–*S*-isomer			
A:PHE156:HN—A:*S*:O1A	2.47766	Hydrogen Bond	Conventional Hydrogen Bond
A:*S*:AH22—A:ASP22:OD1	2.50578	Hydrogen Bond	Conventional Hydrogen Bond
A:*S*:H1A—A:ALA154:O	2.26254	Hydrogen Bond	Conventional Hydrogen Bond
A:GLY48:CA—A:*S*:Br1A	3.50608	Hydrogen Bond	Carbon Hydrogen Bond
A:VAL49—A:*S*	5.34722	Hydrophobic	Alkyl
A:*S*:Br1A—A:ILE131	3.98909	Hydrophobic	Alkyl
A:PHE156—A:*S*	4.95085	Hydrophobic	π∙∙∙Alkyl
A:*S*—A:LEU126	5.23168	Hydrophobic	π∙∙∙Alkyl
A:*S*—A:ALA129	4.51865	Hydrophobic	π∙∙∙Alkyl
A:*S*—A:VAL155	4.75342	Hydrophobic	π∙∙∙Alkyl

**Table 8:** Amino acid residues with the strongest fluctuations in complexes of the *R*-isomer of **1** with Papain-like protease (PLpro) and nonstructural protein 3 (Nsp3_range 207–379-AMP), and the complex of the *S*-isomer of **1** with nonstructural protein 3 (Nsp3_range 207–379-AMP).

PLpro–*R*-Isomer	Nsp3_Range 207–379-AMP–*R*-Isomer	Nsp3_Range 207–379-AMP–*S*-Isomer
ARG0	VAL3	VAL3
GLU1	TYR42	ASN4
VAL2	LEU43	TYR42
ARG3	LYS44	LYS44
THR4	HIS45	ASN59
LYS6	GLY46	ASN72
ASP22	GLY47	LYS76
MET23	GLY48	PRO98
SER24	ALA50	ASN99
MET25	LYS55	VAL100
GLN29	ASN58	ASN101
HIS47	ALA70	LYS102
ASN48	THR71	GLY103
SER49	ASN72	GLU104
GLU51	GLY73	GLN107
LYS190	PRO74	ILE131
THR191	LEU75	PHE132
ILE314	LYS102	ARG148
LYS315	HIS119	PHE156
PRO316	GLU170	GLU170

## Data Availability

All the data supporting the conclusions is included within the manuscript and is available on request from the corresponding authors.
